# Do we need internal medicine specialists in physical therapy? Recognizing the need for updating the clinical practice paradigm

**DOI:** 10.3389/fresc.2025.1656054

**Published:** 2025-09-09

**Authors:** Mansour M. Alotaibi

**Affiliations:** ^1^ Department of Rehabilitation, Faculty of Applied Medical Sciences, Northern Border University, Arar, Saudi Arabia; ^2^ Center for Health Research, Northern Border University, Arar, Saudi Arabia

**Keywords:** physical therapy, internal medicine, chronic disease management, exercise therapy, rehabilitation

## Abstract

The growing demand for healthcare services and development of healthcare present an opportunity for expanding physical therapy roles into internal medicine. This perspective discusses the potential benefits and limitations of establishing a formal internal medicine specialization for physical therapists (PTs). While PTs already contribute significantly to chronic disease prevention and treatment, their current scope of practice lacks structured training in internal medicine domains such as metabolic, autoimmune, renal, and systemic inflammatory disorders. Integrating internal medicine into PT education and clinical practice could enhance early identification of red flags, embrace interdisciplinary collaboration, and improve non-pharmacological interventions for various internal medicine-related diseases. Nevertheless, this expansion must be approached with caution, ensuring clear scope definitions, adequate training, and collaborative implementation to mitigate risks such as role ambiguity or misdiagnosis. Drawing on global experiences from advanced practice models and emerging literature, this paper calls for a discussion on the feasibility, safety, and value of internal medicine specialization in physical therapy practice. The goal of this perspective is not to replace medical professionals but to augment chronic disease management through targeted evidence-based rehabilitation strategies and preventative approaches.

## Introduction

Clinical physical therapy practice in the United States allows practitioners to pursue various specialty certifications to address a wide range of diagnoses, including orthopedic, neurological, cardiopulmonary, and pediatric conditions (1). Physical therapists (PTs) play a pivotal role in providing exercise interventions across multiple domains, including musculoskeletal, neurological, cardiopulmonary, and oncology, primarily to enhance movement and overall well-being (2). In the United States, all states offer some degree of direct access to PTs without physician referrals (unrestricted access in 21 states and access with provisions in 29 states as of July 2024) (3), necessitating a high level of diagnostic proficiency among PTs. This model has demonstrated significant advantages, including increased convenience, reduced wait times, and decreased healthcare-related costs (4).

As the global population continues to age, the demand for geriatric physical therapy services has increased. Older adults frequently exhibit complex health conditions that extend beyond musculoskeletal impairments, requiring a more integrated and interdisciplinary approach to care. Current physical therapy scopes of practice address various patient needs, including geriatric, orthopedics, neurology, cardiopulmonary care, and wound management; however, none specifically focus on internal medicine. Given the high prevalence of multimorbidity in aging populations, this gap in specialization presents a missed opportunity to enhance patient care.

Although PTs are well-trained to screen for systemic diseases, certain internal medicine conditions present with symptoms that overlap with musculoskeletal disorders, increasing the risk of misdiagnosis. The “Big Three” conditions, vascular diseases, infections, and cancer, can mimic common orthopedic complaints, underscoring the importance of specialized training (5) For instance, acute myocardial infarctions (AMI) may manifest as shoulder pain, and a failure to recognize this subtle presentation could lead to life-threatening delays in care (6). A prospective multicenter study found that approximately 18% of AMI patients experienced diagnostic inconsistencies (6). Given that shoulder pain can mimic symptom of AMI, PTs must possess the clinical expertise needed to accurately distinguish musculoskeletal issues from potentially life-threatening conditions (7). While foundational physical therapy education provides initial training in red flag identification, these complexities warrant the need for a formal credential in internal medicine. Establishing such a pathway would improve diagnostic accuracy and contribute to patient safety, especially in a direct-access environment, where PTs serve as first contact providers.

Physical therapy is uniquely positioned to deliver non-pharmacological interventions, including exercise, manual therapy, and modalities, into holistic patient care. Providing PTs with structured training in internal medicine would enhance their understanding of human pathology and physiology, enhancing their diagnostic capabilities and treatment efficacy. In addition, several chronic diseases may benefit from physical therapy, and patients of such diseases often do not consider PTs part of their healthcare providers. For example, patients with diabetic gastroparesis would rarely seek PTs to improve their symptoms, as management is traditionally led by physicians using dietary and pharmacological interventions. Alongside pharmacological treatment, PTs could help provide exercise intervention and education for such patients to augment medical management and enhance patient outcomes (8). These contributions would occur in coordination with medical management and within clearly defined professional boundaries. The inclusion of specialized PTs in internal medicine in the multidisciplinary care teams would enhance the awareness of PTs’ roles in these conditions and expand accessibility to non-pharmacological support, thereby enhancing patients’ outcomes.

It is important to acknowledge that PTs already play an essential role in the management of many internal medicine-related conditions within the domains of cardiopulmonary, geriatric, and primary care. These contributions are exemplified in management of conditions such as chronic obstructive pulmonary disease (9, 10), heart failure (11, 12), osteoporosis (13), and diabetes (14, 15). However, the proposed internal medicine specialization would expand the scope beyond the above-mentioned domains to encompass systemic metabolic, renal, autoimmune, hematologic, and inflammatory diseases, where PTs currently have limited formal training and recognition. Nevertheless, the overlap between the proposed internal medicine specialty and existing recognized specialties is inevitable. Developing clear scope of practice guidelines, competency standards, and certification pathways would be essential to minimize this overlap and enhance clinical roles and professional development pathways.

Expanding the scope of physical therapy into domains traditionally managed by physicians and nurse practitioners, such as chronic disease management and systemic risk screening may raise concerns about role encroachment or redundancy. Thus, the integration of an internal medicine training into physical therapy practice must be approached with sensitivity to the dynamics of the broader healthcare team. Regardless, the intent of developing this specialization for PTs is to reinforce rather than to replace existing medical roles. Internal medicine-specialized PTs, once integrated in the healthcare system, would operate within clearly defined clinical boundaries, focusing more on non-pharmacological interventions, early risk identification, functional optimization, and interdisciplinary healthcare planning. Evidence suggests that PTs with advanced training working in primary care settings and emergency departments demonstrated high-level of diagnostic accuracy in line with physicians’ diagnosis with musculoskeletal cases (16). PTs with specific training in internal medicine would not assume responsibility for diagnosing complex internal medicine conditions but would serve as collaborators in early identification and secondary prevention. Thus, PTs could successfully function as physician extenders, as suggested by experience from primary care (17) and military setting (18). Traditionally, PTs have successfully served as physician extenders in military settings, handling over 3,500 visits within direct-access models (19). This model has demonstrated benefits such as decreased healthcare expenditure and fewer lost duty days due to musculoskeletal injuries. Expanding this model to include internal medicine training could further elevate the role of PTs in comprehensive patient management and preventative care (17).

This perspective paper advocates for the establishment of an Internal Medicine Clinical Specialist certification within geriatric physical therapy practice. The addition of internal medicine training could elevate PTs’ role in chronic disease prevention and management. By providing PTs with specialized training in internal medicine, they can better contribute to the early detection, management, and prevention of systemic diseases in diverse populations, including older adults. For example, exercise therapy has been shown to significantly improve glycemic control in individuals with type II diabetes mellitus (T2DM) (20, 21). However, most DM patients seek physical therapy services only for musculoskeletal complaints, despite evidence indicating that approximately 80% of physical therapy referrals involve patients with prediabetes or diabetes (22). Internal medicine training would enable PTs to identify T2DM risk factors early, predict complications (23), and contribute to comprehensive care plans that incorporate exercise and lifestyle modifications with pharmacological treatment. This opportunity would allow PTs to further contribute to the provision of healthcare and improve the quality of healthcare services.

Training in internal medicine for PTs would also benefit patients with autoimmune disorders, such as celiac disease, which is associated with reduced bone mineral density (BMD) due to impaired nutrient absorption (24). Individuals with celiac disease face a 50% increased risk of fractures, yet current PT education does not emphasize targeted rehabilitation strategies for this population (25). Limited evidence suggests that physical therapy interventions may improve BMD in this population (26). While adherence to a gluten-free diet with or without pharmacological intervention would recover the diminished BMD at 5 years (27), PTs can further help in speeding up the recovery by providing tailored exercise interventions individuals with celiac disease (28), thereby reducing the risks for skeletal fractures. Additionally, mitochondrial dysfunction plays a critical role in conditions such as exertional rhabdomyolysis (29, 30), a serious consequence of extreme physical exertion commonly seen in military personnel and athletes. PTs are well-trained in providing education and treatment for individuals with this condition (31), and, if they receive specialized training in internal medicine, can further help these individuals improve their well-being.

From an economic perspective, reimbursement challenges often hinder access to PT services for patients with internal medicine-related conditions (32). While evidence on this topic is limited, insurance providers frequently deny coverage due to the lack of clear diagnostic justification for PT intervention. Establishing an internal medicine specialty within PT practice would create a distinct healthcare segment, reinforcing the necessity of PT services in managing internal medicine conditions. Well-trained PTs in internal medicine could contribute to healthcare cost reduction by preventing unnecessary hospitalizations and surgical interventions, thereby alleviating financial burdens on both patients and healthcare systems (33). Preliminary evidence suggests that physical therapy interventions via direct access compared to referred episodes can reduce healthcare costs by lowering hospital admissions, reducing medication use, and improving patient outcomes (4, 33). Nonetheless, robust designs, such as large-scale longitudinal studies and clinical trials specifically address the effects of physical therapy on internal medicine-related conditions. Therefore, future research should examine efficacy, cost-effectiveness, and feasibility of the proposed internal medicine in physical therapy model across different health systems.

Although PTs are increasingly involved in the management of chronic medical conditions, there is no formally recognized credential in internal medicine for PTs. In the United States, the American Board of Physical Therapy Specialties offers 11 specialty certifications, yet none focus on internal medicine (Table 1). In the United Kingdom, specialized physical therapy practice roles require meeting the standards of receiving adequate training and whether the activities PTs practice fall within the general scope of practice of the profession to perform the activity effectively and safely (34). The Charted Society of Physiotherapy participates in the world physiotherapy recognized 13 specialty groups, none of which include internal medicine (35). Similarly, the Australian Physiotherapy Association recognizes nine fields of practice. However, internal medicine is none of the recognized specialty programs available for PTs (36). Establishing a structured specialty in internal medicine in PT would expand the scope of practice under a standardized competency framework and enhance quality of care across healthcare systems.

**Table 1:** Summary of the currently existing specialty programs for physical therapists and the proposed internal medicine specialty.

Specialty	Description
Cardiovascular and pulmonary	Focuses on physical function of cardiopulmonary system, specifically with those who have cardiopulmonary dysfunction
Acute care	Provide care for people with urgent medical conditions who need immediate medical attention
Clinical electrophysiology	Includes the use of electricity to monitor, measure, or produce physiological responses to evaluate, treat, and prevent human dysfunction
Geriatrics	Covers the needs of older adults and aging-related physical dysfunction
Neurology	Provides evaluation and treatment to individuals with movement problems caused by neurological disease or trauma
Oncology	Involves physical therapy practice to maximize movement and well-being to individuals affected by cancer
Orthopedics	Provides care to the musculoskeletal system that involves bones, muscles, joint, tendons, and other soft tissues following an injury
Pediatrics	Helps improve range of motion, strength, flexibility, and movement pattern in children affected by various diagnoses and injuries
Sports	Works with athletes to restore function, mobility, and strength following sport-related injury
Women's health	Provides expert treatment for pelvic floor dysfunction and obstetrical care
Wound management	Delivers wound care to patients in hospitals and outpatient setting utilizing wound care protocols and using unique therapeutic modalities
Internal medicine	Allows expert to identify early signs of systemic diseases, implement targeted interventions, and enhance patient outcomes through comprehensive, multidisciplinary care

Introducing an internal medicine specialty for PT could support improved chronic disease treatment and interprofessional care coordination. By integrating internal medicine principles into PT education and clinical training, PTs can play a more significant role in chronic disease care, improved diagnostic accuracy, and enhanced interdisciplinary collaboration. This initiative has the potential to improve patient care, strengthen interdisciplinary care and improve healthcare system efficiency. Given the mounting evidence supporting the benefits of exercise in managing internal medicine conditions (2, 20, 37), the time has come to formalize this specialization and expand the scope of PT practice to meet the evolving needs of modern healthcare.

While the potential benefits of internal medicine specialization for PTs are compelling, it is critical to consider potential limitations, particularly in terms of resource allocation. If implemented without adequate governance and stakeholder alignment, this model of role expansion could result in scope confusion, inefficiencies, or resistance from other professionals. Although PT in general has been shown to reduce hospitalization rates and prevent costly complications in patients with chronic diseases (38, 39), there is limited direct economic evidence supporting the cost-effectiveness of internal medicine-certified PTs (39). Thus, the specific economic impact of internal medicine specialization in PT warrants further investigation. Implementing such a program would require capital investment in curriculum development, training pathways, certification frameworks, and interdisciplinary integration, and this may be challenging in resource-constrained systems. However, strategic investment in this specialization can be justified through multiple anticipated benefits: improved patient safety through early recognition of red flags, enhanced integration in chronic disease treatment pathways, and decreased healthcare expenditures via reduced complications and unnecessary procedures. Perhaps small pilot programs and cost-effectiveness trials could be conducted to assess the value proposition of this specialization in PT.

As with any proposed expansion of professional roles, the implementation of an internal medicine specialization for PTs may be associated with potential risks. These include role ambiguity, interprofessional resistance, legal liability, and the possibility of fragmented care if integration is not implemented with full coordination. Addressing these risks requires clear scope-of-practice definitions, certification, and interdisciplinary training frameworks for guiding the future steps. Experience from the UK's advanced practice physiotherapy model provides a valuable precedent. Several studies have documented that advanced practice physiotherapy deliver care with diagnostic accuracy comparable to that of physicians in musculoskeletal triage settings (40, 41), decrease wait times (42), enhance patient satisfaction (43, 44), and reduce downstream healthcare costs (45). These outcomes were achieved within structured models that encourage collaboration, training, and risk mitigation. Adapting similar principles could guide the safety and effectiveness for implementing internal medicine specialization in physical therapy.

## Data Availability

The original contributions presented in the study are included in the article/Supplementary Material, further inquiries can be directed to the corresponding author.
